# Impact of Discrimination on Help-Seeking Behavior Among Individuals With Serious Mental Illness in South Korea: Role of Social Participation Services

**DOI:** 10.1007/s10597-025-01458-9

**Published:** 2025-02-25

**Authors:** Subin Na, Sang Kyoung Kahng, Phyllis Solomon

**Affiliations:** 1 School of Social Policy & Practice, University of Pennsylvania, Philadelphia, PA 19104 USA; 2 Department of Social Welfare, Seoul National University, Seoul, Republic of Korea

**Keywords:** Serious mental illness, Discrimination, Help-seeking, Social participation services, South Korea

## Abstract

This study investigated the relationship between perceived discrimination, help-seeking behaviors, and the adequacy of social participation services among individuals with serious mental illness in South Korea. Data were drawn from a 2020 survey conducted by the National Human Rights Commission of Korea, involving 607 participants who used community-based mental health rehabilitation facilities. Structural equation modeling was employed to examine the extent to which perceived discrimination in healthcare, employment, and personal–social relationships affects help-seeking behaviors, including the mediating effect of perceived adequacy of social participation services. Results revealed that experiences of discrimination in healthcare and employment significantly influenced help-seeking, whereas discrimination in personal–social relationships did not. Although the adequacy of social participation services positively impacted help-seeking, it did not mediate the relationship between discrimination and help-seeking behaviors. The findings suggest addressing discrimination in healthcare and employment and expanding diverse, accessible social participation services are crucial for encouraging help-seeking among individuals with serious mental illness across regions in Korea.

## Introduction

The United Nations Convention on the Rights of Persons with Disabilities (UN CRPD) represents a significant milestone in protecting the rights of individuals with mental health conditions or psychosocial disabilities, marking a shift from a medical to a human rights model of disability (Freeman et al., 2015; Gooding, 2017). This human rights approach, now the leading perspective for treating serious mental illness, emphasizes providing comprehensive services that promote various human rights aspects, including employment, education, and social participation (National Human Rights Commission of Korea, 2021; UN, 2007). While South Korea ratified the CRPD in 2008, there is a widespread view that the country has not made sufficient strides in aligning its mental health service framework with the international human rights standards established by the convention (Lee et al., 2021; National Human Rights Commission of Korea, 2020).

One of the most significant barriers to progress is the persistent discrimination faced by individuals with serious mental illness, which contributes to stigma and social exclusion. Numerous studies highlight that this group experiences more severe forms of social discrimination than other disability groups, across personal and institutional spheres including interpersonal relationships, education, employment, healthcare, legal policies, and community integration (Je, 2021; Kahng et al., 2021; National Center for Mental Health, 2024; National Human Rights Commission of Korea, 2018, 2021; Song et al., 2018). Studies show that Koreans with serious mental illness face exceptionally high levels of social stigma and discrimination, experiencing the most severe forms among all disability groups, with their social exclusion levels being six to ten times higher than those with other disabilities (Oh, 2018; Park & Lee, 2016).

Proactive coping strategies, such as help-seeking behaviors, are crucial for overcoming the challenges posed by discrimination, and these strategies align with the human rights model’s view of individuals as agents in their own recovery (National Human Rights Commission of Korea, 2020). However, research on help-seeking behaviors of individuals with serious mental illness, particularly in Korea, remains sparse. Given that these behaviors are shaped by the surrounding social environment (Kahng, 2018), the study adopts the social model of disability, which frames disability not as an individual characteristic but as a consequence of societal barriers (Kwak & Kim, 2004).

Both the human rights model and the social model of disability emphasize the need for services and policies that strengthen individual capacity, while simultaneously addressing societal structures that perpetuate exclusion and marginalization. In this context, social participation services are seen as essential for promoting active coping behaviors. The right to social participation is a fundamental human right, and these services can play a pivotal role in empowering individuals and supporting their recovery (Moon et al., 2020; Youn, 2021). Despite their significance, research on the availability and impact of social participation services for individuals with psychiatric disabilities remains limited, both domestically and internationally.

The present study aimed to explore the relationship between experiences of discrimination, help-seeking behaviors, and the perceived adequacy of social participation services for individuals with serious mental illness. Specifically, we investigated the mediating role of social participation services in the relationship between discrimination experiences and help-seeking behaviors among individuals with serious mental illness. By focusing on whether these services can mitigate the effects of discrimination and foster help-seeking, this study provides valuable empirical evidence to inform future policy development and service provision. Additionally, by employing the stress appraisal-coping model (Lazarus & Folkman, 1984), the research sought to deepen the understanding of individual differences in responses to discrimination, particularly whether social participation services can act as a crucial mechanism in promoting recovery.

## Background and Significance

### Discrimination Against Individuals With Serious Mental Illness in Korea

Stigma arises when elements of labeling, stereotyping, separation, status loss, and discrimination converge within a context of power dynamics that enable their perpetuation (Link & Phelan, 2001). Discrimination, as the behavioral manifestation of stigma, can be categorized into enacted and perceived discrimination. Enacted discrimination involves direct experiences of social discrimination in areas such as employment, housing, healthcare, and interpersonal relationships, while perceived discrimination refers to individuals’ subjective interpretations of their experiences as discriminatory, regardless of whether such actions were intended or occurred (Jeon, 2009; Link, 1987). Stigma and discrimination are significant sources of stress for individuals with serious mental illness and are among the greatest barriers to recovery (Rüsch et al., 2009; Wahl, 2012). According to Deegan (2000), what is truly disabling to individuals with serious mental illness is stigma that is widespread in the general population, rather than the psychotic symptoms themselves.

In South Korea, stigma and discrimination toward mental illness remain prevalent, as revealed by a 2024 survey conducted by the National Mental Health Center, targeting 3,000 members of the general public aged between 15 and 59. The survey showed that 50.7% of respondents believed their friends would distance themselves if they were diagnosed with a mental illness, and 64.6% viewed individuals with mental illness as more dangerous than those without (National Center for Mental Health, 2024). Both responses represent an increase from 2022, indicating that deeply ingrained social stigma and misconceptions not only persist, but may be worsening.

Previous research also shows that individuals with psychiatric disabilities encounter pervasive stigma from society and face discrimination across many aspects of their lives (Thornicroft, 2006). Studies have identified discrimination in areas such as access to social services, healthcare, education, employment, certification, voting rights, media, culture, interpersonal relationships, public transportation, and public facilities (Kahng et al., 2021; National Human Rights Commission of Korea, 2021). This discrimination extends beyond individual experiences to institutional and societal levels, affecting nearly every aspect of life, with individuals with psychiatric disabilities perceiving the highest levels of discrimination compared to other disability groups (Oh, 2018). While existing research in Korea has primarily focused on documenting the severity and negative consequences of discrimination (Kim & Yeum, 2013; Lee & Kim, 2024; Song et al., 2018), less attention has been given to the strengths and capacities of Koreans with serious mental illness in addressing these challenges. Therefore, this study sought to shift the focus to the active, self-directed efforts of individuals with psychiatric disabilities in combating and rectifying discrimination, positioning them as agents of change rather than passive subjects affected by stigma and discrimination.

### Help-Seeking Behavior Among Individuals With Serious Mental Illness

Research on help-seeking behavior has been conducted across various fields, including counseling, education, and social work practice. Traditionally, help-seeking refers to seeking professional assistance, such as therapy or psychiatric services, rather than informal support from friends or acquaintances (Fischer & Turner, 1970). In the context of mental health, help-seeking behavior can be understood narrowly as the utilization of services (Hom et al., 2015) or more broadly as seeking assistance from a range of sources, including mental health professionals, healthcare providers, religious leaders, educators, support groups, or family and friends (Horwitz, 1977; Rogler & Cortes, 1993). Building on the broad definition of help-seeking, this study conceptualized help-seeking behavior as the act of individuals with serious mental illness to seek help from formal and/or informal systems to address discrimination. According to Lazarus and Folkman (1984), the theoretical framework on which this study is based, help-seeking behavior is regarded as a form of problem-focused coping, as it involves actively attempting to resolve discriminatory situations by changing the environmental conditions that contribute to personal stress.

In studies examining the relationship between help-seeking behavior and discrimination, Scott and House (2005) argued that the more discrimination a person experiences, the less likely they are to resolve the problem and control the situation. As a result, they tend to rely more heavily on emotion-focused coping mechanisms, such as regulating their emotions internally through avoidance or denial. Other studies have also shown that, among Black and Mexican-origin adolescents, the accumulation of discriminatory experiences tends to lead to passive, emotion-focused coping strategies, such as compliance and avoidance (Compas, 1995; Brittian et al., 2013). Given that increased exposure to discrimination is associated with passive coping strategies, we hypothesize that individuals with serious mental illness who experience higher levels of discrimination may exhibit fewer active help-seeking behaviors.

### Social Participation Service for Individuals With Serious Mental Illness in Korea

Social participation is crucial for people with disabilities, as it fulfills their need for relationships, recognition, and societal adaptation, while helping them escape isolation, enhance self-efficacy, and improve quality of life (Moon et al., 2020). From a policy perspective, mental health services grounded in the human rights model need to encompass a comprehensive array of supports that promote community integration, including employment, leisure activities, housing, education, independent living, and social participation (National Human Rights Commission of Korea, 2020). However, research on these services in Korea remains limited, particularly regarding their role in supporting the recovery of individuals with psychiatric disabilities (Webber & Fendt-Newlin, 2017).

Building on previous research, this study defines social participation services as those supporting individuals with serious mental illness in leading independent and autonomous lives through various social and cultural activities, while focusing on the extent to which individuals perceive the adequacy of these services. Research has consistently demonstrated that perceived support has a stronger influence on well-being and stress management than the actual support received (Helgeson, 1993; Wethington & Kessler, 1986). This distinction is particularly relevant in the context of social participation services, as how individuals interpret and evaluate the availability and quality of these services can shape their willingness to engage with them and influence their overall recovery process.

Social participation services may serve as a crucial mechanism through which individuals mitigate the negative effects of discrimination on help-seeking behaviors. Discrimination can lead to feelings of exclusion, diminished self-worth, and internalized stigma, which reduce individuals’ motivation and willingness to seek help (Corrigan & Kosyluk, 2014). According to the stress appraisal-coping model (Lazarus & Folkman, 1984), individuals facing stressors may appraise these experiences as threats that exceed their personal coping resources, leading to passive coping strategies such as avoidance. However, perceiving social participation services as adequate and supportive may buffer these negative effects by providing external resources that enhance individuals’ coping capacity and cognitive appraisal on these experiences. These services can foster a sense of belonging, reinforce self-efficacy, and promote subjective recovery, thereby increasing individuals’ confidence in seeking help, despite discriminatory experiences (Thomas et al., 2016). In this way, the perceived adequacy of social participation services would function as a connector. While discrimination may initially reduce the likelihood of help-seeking, perceiving these services as accessible and supportive can reframe individuals’ coping appraisals and encourage proactive help-seeking behaviors.

Thus, this study examines the mediating role of perceived adequacy of social participation services, conceptualized as a form of social support, in the relationship between discrimination experiences and help-seeking behaviors. Specifically, we hypothesize that individuals with greater experiences of discrimination may be less likely to seek help. However, if they perceive social participation services as adequate and supportive, these services may counteract the negative impact of discrimination and facilitate help-seeking behaviors. By focusing on the perceived adequacy of these services, this study seeks to highlight their role as a potential recovery resource in mitigating the adverse consequences of discrimination for individuals with serious mental illness.

### Stress Appraisal-Coping Model

The theoretical framework for this study is Lazarus and Folkman’s (1984) stress appraisal-coping model, which consists of five key factors: causal antecedents, stress, cognitive appraisal, coping, and adaptation. Lazarus and Folkman (1984) proposed that the relationships among these factors are not linear but circular and interconnected through feedback loops. Building on this framework, this analysis sought to examine the extent to which individuals with serious mental illness experience stress through discrimination, the extent of their appraisal of the adequacy of social participation services available to them, and the extent they engage in help-seeking behavior to manage discriminatory situations.

Specifically, stress refers to a relationship between an individual and their environment that is perceived as threatening to the person’s well-being by exceeding their available resources (Lazarus & Folkman, 1984). In this study, discrimination experienced by individuals with serious mental illness, can be understood as a source of stress. Stigma and discrimination are generally perceived as stressors (Link & Hatzenbuehler, 2016), particularly for individuals with serious mental illness who frequently experience social discrimination (Corrigan, 2005; Hinshaw, 2007; Link & Phelan, 2001; Rüsch et al., 2009).

However, according to Lazarus and Folkman (1984), stress is not imposed on individuals in a one-directional way by the external environment, and the same stimulus may not be perceived as stressful by everyone. To explain these individual differences, Lazarus and Folkman (1984) introduced the concept of “cognitive appraisal,” referring to how individuals assess stressful situations based on their available resources. Cognitive appraisal consists of two interrelated stages: primary appraisal, where individuals evaluate whether an event is challenging, threatening, or irrelevant, and secondary appraisal, where they assess their ability to cope with the situation. In this study, the adequacy of social participation services relates to secondary appraisal. For instance, if individuals with serious mental illness perceive that society provides sufficient social participation services, they may feel they have necessary and sufficient support and resources to address discrimination. Conversely, if they perceive these services as insufficient, they may feel they lack the necessary resources to cope with discrimination.

Coping refers to cognitive and behavioral efforts to manage demands perceived as threatening or exceeding personal resources (Lazarus & Folkman, 1984). Coping can be divided into two categories: emotion-focused coping, which seeks to regulate emotions arising from stress, and problem-focused coping, which involves efforts to change the environmental conditions causing stress (Folkman, 1984). In this study, the help-seeking behavior is conceived of as a form of problem-focused coping, given it involves actively seeking to alter the conditions of discrimination. Help-seeking is more likely when individuals perceive the environment as changeable, while emotion-focused coping is more common when they feel the environment cannot be altered (Folkman & Lazarus, 1980).

In summary, the extent to which individuals with serious mental illness engage in help-seeking behavior is influenced by their cognitive appraisal. In this study’s model, it is hypothesized that experiencing discrimination prompts individuals to cognitively appraise the adequacy of social participation services, and this appraisal, in turn, influences their use of problem-focused coping, such as help-seeking behavior. In other words, the adequacy of social participation services may well mediate the relationship between perceived discrimination and help-seeking behaviors among individuals with serious mental illness.

## Method

### Sample and Data Collection

The present study analyzed data from “A Survey on the Mental Rehabilitation Facilities and Human Rights of Users,” conducted by the National Human Rights Commission of Korea in 2020. This survey was carried out to evaluate the performance of mental health rehabilitation facilities, assess the needs of users, and gather basic data to provide appropriate programs for future users (National Human Rights Commission of Korea, 2020). Data were collected between August and November 2020 from individuals with psychiatric disabilities who had utilized community-based mental health rehabilitation facilities, such as residential mental health rehabilitation facilities and community psychiatric rehabilitation centers. A total of 607 participants constituted the study sample. The survey design aimed to gather responses from 300 residents of residential mental health rehabilitation facilities and 300 users of community psychiatric rehabilitation centers. For sample selection, proportional probability sampling was used based on region and facility type. In cases where proportional probability sampling was practically difficult, convenience sampling was employed for the available regions and facility types (National Human Rights Commission of Korea, 2020).

As shown in Table 1, respondents were predominantly male (57.0%; n = 346), while 43.0% (n = 261) were female. Nearly 75% (n = 454) reported a diagnosis of schizophrenia or schizoaffective disorder, while 25.2% (n = 153) were diagnosed with other conditions, including bipolar disorder or major depression. Most participants (92.6%; n = 562) had a high school diploma or higher, whereas only 7.4% (n = 45) had less than a high school education. The majority (93.5%; n = 567) were single, and 42.7% (n = 259) resided in rural areas, while 57.3% (n = 348) lived in urban locations. Over half (55.1%; n = 334) of the participants were recipients of the National Basic Livelihood Security System. Facility types were nearly evenly split, with 49.9% (n = 303) residing in residential psychiatric treatment facilities and 50.1% (n = 304) attending community psychiatric rehabilitation centers. The average age of participants was 42.65 years, and the mean duration of illness was 16.48 years.

**Table 1:** Demographic characteristics of sample (*N* = 607)

	M
Age	42.65 years
Duration of illness	16.48 years

	*n*	%
Gender		
Male	346	57.0
Female	261	43.0
Diagnosis		
Schizophrenia/Schizoaffective disorder	454	74.8
Other (i.e., Bipolar, Major Depression)	153	25.2
Educational level		
High school attendance or less	45	7.4
High school diploma or more	562	92.6
Marital status		
Married or Cohabited	40	6.5
Single	567	93.5
Residence		
Rural Area	259	42.7
Urban Area	348	57.3
National Basic Livelihood Security System (Income Proxy)		
Recipient	334	55.1
Nonrecipient	273	44.9
Facility Type		
Residential Psychiatric Treatment Facility	303	49.9
Community Psychiatric Rehabilitation Center	304	50.1

### Measures

Perceived discrimination, which was originally measured in the survey using 26 items from the ‘Areas of Experience of Discrimination Due to Mental Illness,’ a list designed to assess individuals with serious mental illness and their perceptions of discrimination in various areas while living in the community. For each area listed, participants indicated whether they perceived discrimination (i.e., “Yes” or “No”). However, since the measurements were not standardized, we decided to use only a selection of the items to measure the concept, with the selection criteria based on variance. We checked the variances for each item and removed those with a variance lower than 0.1, as variables with low variance provide less information and make it harder for models to estimate relationships, weakening the overall analysis (Bollen, 2014). Therefore, 13 items remained and were selected for use (See Table 5 for the selected items). The internal consistency of the assessment was evaluated using the Kuder–Richardson Formula 20 (KR-20), which is suitable for dichotomous items. The KR-20 value for the scale was 0.782, indicating good internal consistency.

Help-seeking was measured using 13 items that investigated whether or not respondents sought help after answering “Yes” to any of the 13 questions of perceived discrimination. For each area indicating yes to discrimination encountered, participants answered whether they sought help. In this study, respondents’ answers were coded as ‘0 = no help-seeking’ and ‘1 = help-seeking,’ and the scores for each question were summed for a total score. The KR-20 reliability value for the scale was 0.829, indicating good internal consistency.

The adequacy of social participation services was measured using 15 items from ‘Services for Promoting Social Participation of Individuals with Serious Mental Illness,’ a list of services implemented in Korea to support the social participation of individuals with serious mental illness (See Table 2). For each service listed, respondents were asked to indicate the extent to which they perceived the service as being adequately provided. Each item was rated on a 4-point Likert-type scale (i.e., 0 = very dissatisfied, 1 = dissatisfied, 2 = satisfied, 3 = very satisfied). We checked the variances for each item using 0.9 as the cutoff for low variance, but all items had variances above this threshold. Cronbach’s α assessing internal consistency was 0.986.

**Table 2:** List of ‘services for promoting social participation of individuals with SMI’

Variable	Items
Adequacy of social participation services	1. Social Skills Training Programs 2. Self-Help Group Services 3. Peer Support Specialists Training Programs 4. Art and Cultural Activity Programs 5. Leisure and Recreational Activity Programs 6. Physical Activity and Wellness Programs 7. Community Engagement and Volunteer Programs 8. Support for Participation in Public Awareness Campaigns 9. Support for Establishing and Participating in Peer-Led Organizations 10. Public Guardianship Services for Supported Decision-Making 11. Support for Involvement in Service and Program Development 12. Support for Involvement in Service and Program Evaluation 13. Support for Participation in Reviewing and Revising Facility Policies 14. Human Rights Education and Training Programs 15. Support for Participation in Human Rights Advocacy and Monitoring Activities (e.g., Human Rights Monitoring Group)

### Statistical Analyses

The statistical analyses were conducted using R. Prior to the analysis, the data were screened for outliers and errors. To handle missing data, multiple imputation was performed using the ‘mice’ package in R, following the procedure outlined by Enders (2010). Before running the imputation, the data were assessed to ensure that the missing values were missing at random (MAR). Additionally, the level of missing data was analyzed, confirming that less than 10% of the data was missing across the scale scores. The assumption of MAR was met, and the overall rate of missing data across scales was acceptable. Descriptive analyses were conducted to describe the sample characteristics. Correlation analyses were then used to identify associations between the continuous variables.

The hypothesized mediation model was analyzed using a two-step approach (Anderson & Gerbing, 1988) with the ‘lavaan’ package in R. In the first step, a confirmatory factor analysis (CFA) was performed to create a measurement model with a good fit to the data. This CFA included three latent variables and 13 observed variables. In the second step, structural equation modeling (SEM) was employed to test the hypothesized relationships between the three domains of discrimination and help-seeking behavior, as well as the mediating role of adequacy of social participation services in this relationship. According to Westland (2010), a minimum sample size of N = 200 is required to detect medium-sized relationships with 80% power, based on the ratio of observed variables to latent variables. Given that the sample size was 607, we concluded that there was sufficient power to test the hypotheses. Model fit was evaluated using chi-squared tests and a set of recommended fit indices: the Comparative Fit Index (CFI) where values of ≥ 0.90 indicating a reasonable model fit (Brown, 2014; Kline, 2005), the Root Mean Square Error of Approximation (RMSEA), with values ≤ 0.05 indicating a good fit and values between 0.05 and 0.08 suggesting an adequate fit (Brown, 2014; Hu & Bentler, 1999).

## Results

### Preliminary Analyses

Descriptive statistics for the main variables are presented in Table 3. The mean score for perceived discrimination was 3.23 (SD = 3.15) with skewness of 0.893 and kurtosis of − 0.051, both within the acceptable range of ± 2 (George & Mallery, 2010), indicating a relatively normal distribution. For discrimination in healthcare services, the mean score was 1.80 (SD = 1.86), with skewness of 0.656 and kurtosis of − 0.812, suggesting a slightly positively skew, but otherwise normal distribution. Employment and job-related discrimination had a mean score of 0.99 (SD = 1.47), skewness of 1.387, and kurtosis of 0.736, indicating a moderate skew, but still acceptable distribution. Discrimination in personal–social relationships had a lower mean of 0.43 (SD = 0.66), with skewness of 1.261 and kurtosis of 0.294, both well within acceptable ranges, indicating moderate positive skewness. The help-seeking variable had a mean score of 1.31 (SD = 2.23) with skewness of 2.356 and kurtosis of 1.946, indicating a high positive skewness that is slightly above the acceptable threshold of 2. Lastly, perceived adequacy of social participation services had a mean score of 13.38 (SD = 6.89), with skewness of − 0.026 and kurtosis of − 0.486, both of which fall within the acceptable range, indicating a relatively normal distribution. Additionally, bivariate correlations revealed statistically significant relationships among all the variables, as shown in Table 4.

**Table 3:** Descriptive statistics for main variables

Variables	Min	Max	*M*	*SD*	Skewness	Kurtosis
1. Perceived Discrimination	0	13	3.23	3.15	0.893	− 0.051
1-1. Discrimination in Healthcare Services	0	6	1.80	1.86	0.656	− 0.812
1-2. Employment and Job-Related Discrimination	0	5	0.99	1.47	1.387	0.736
1-3. Discrimination in Personal–Social Relationships	0	2	0.43	0.66	1.261	0.294
2. Help-Seeking	0	12	1.31	2.23	2.356	1.946
3. Perceived Adequacy of Social Participation Services	0	27	13.38	6.89	− 0.026	− 0.486

**Table 4:** Pearson correlations for main continuous variables

Variables	1-1	1-2	1-3	2	3
Discrimination in Healthcare Services	1				
Employment and Job-Related Discrimination	0.4196***	1			
Discrimination in Personal–Social Relationships	0.2594***	0.4597***	1		
Help-Seeking	0.5969***	0.5168*	0.3576***	1	
Perceived Adequacy of Social Participation Services	− 0.0735*	− 0.1324**	− 0.1028*	− 0.0215*	1

Note: *p < 0.05, **p < 0.01, *** p < 0.001

#### Testing the Measurement Model

A CFA was conducted to assess the three hypothesized latent categories: (1) discrimination in healthcare services, (2) employment and job-related discrimination, and (3) discrimination in personal–social relationships. The measurement model and corresponding factor loadings are presented in Table 5. The initial model fit statistics were: χ^2^(62) = 330.864 (p < 0.001), CFI = 0.887, and RMSEA = 0.085 (90% CI 0.076–0.094). Although these results suggest an acceptable fit, there remained potential for further improvement.

**Table 5:** Results of confirmatory factor analysis

	Factor loadings	
	Initial model (N = 607)	Improved model (N = 607)
Discrimination in Healthcare Services		
I was unable to be discharged from the hospital when I wanted to because of my mental illness	0.719	0.720
My opinion was ignored when making treatment decisions because of my mental illness	0.776	0.776
There were changes to the amount of antipsychotic drugs administered, without any consultation or input from me	0.634	0.635
I was physically restrained without explanation because of my mental illness	0.676	0.676
I was unable to be admitted to a community mental health facility due to the severity of my psychotic symptoms	0.439	0.437
My discharge from the psychiatric hospital was delayed because there was no place for me to live because of my mental illness	0.401	0.403
Employment and Job-Related Discrimination		
I could obtain a job because of my mental illness	0.698	0.687
I was dismissed from my job because of my mental illness	0.770	0.784
I could not be promoted because of my mental illness	0.782	0.802
I have been underpaid because of my mental illness	0.692	0.676
I was unable to acquire job-related licenses due to my mental illness (e.g., restrictions or prohibitions on obtaining qualifications)	0.430	
Discrimination in Personal–Social Relationships		
I was bullied at school because of my mental illness	0.613	0.622
I was discriminated against in dating, marriage, and childbirth due to my mental illness	0.509	0.502
Model Fit	χ^2^(df) = 330.864(62) CFI = 0.887 RMSEA = 0.085 (0.076–0.094)	χ^2^(df) = 242.905 (51) CFI = 0.913 RMSEA = 0.079 (0.069–0.089)

Following the initial CFA, one item from the employment discrimination group (“I was unable to acquire job-related licenses due to my mental illness”) demonstrated a weak factor loading and was subsequently removed from the model. The revised CFA model showed improved fit indices: χ^2^(51) = 242.905 (p < 0.001), CFI = 0.913, and RMSEA = 0.079 (90% CI 0.069–0.089). The enhancements in CFI and RMSEA indicated a better-fitting model. Additionally, all factor loadings were statistically significant (p < 0.01), supporting the convergent validity of the indicators. These results suggest that the latent variables were well represented by their respective indicators. The removal of one item which related to discrimination in the acquisition of job-related licenses was not only supported by the model fit indices results but also conceptually. Unlike the other items that capture ongoing workplace discrimination, Item 18 may reflect a singular event, as individuals who are denied a license are unlikely to repeatedly face such rejections.

### Structural Equation Modeling

**Fig. 1:**
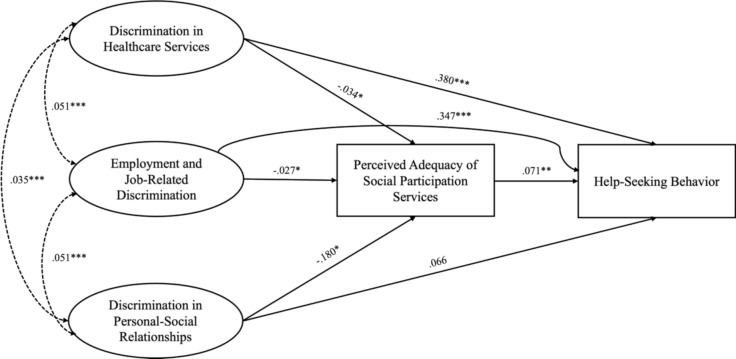
Full path model of the relationship between discrimination, help-seeking behaviors, and the perceived adequacy of social participation services. *Regression paths are represented by straight lines with one arrowhead, while covariances are shown as dotted lines with two arrowheads

After refining the measurement model, SEM was employed to examine the relationships between various forms of discrimination and help-seeking behavior, with social participation services as a mediator. As shown in Fig. 1, the full path model included both direct and mediated paths from the discrimination domains to help-seeking behavior. All three domains of discrimination significantly affected the perceived adequacy of social participation services (p < 0.01), suggesting that individuals with serious mental illness who face more discrimination tend to perceive social participation services as less adequate. Discrimination in healthcare and employment significantly affected help-seeking behavior, whereas discrimination in personal–social relationships did not. Additionally, the effect of perceived adequacy of social participation services on help-seeking behavior was statistically significant (p < 0.05), indicating that individuals with serious mental illness who perceive the adequacy of social participation services to be high are more likely to engage in help-seeking behavior. However, the full path model showed poor fit based on both the CFI (0.834) and RMSEA (0.069), suggesting room for improvement (see Table 6).

**Table 6:** Fit indices for full path model and trimmed model

	Full path model	Trimmed model
χ2 (p)	1839.968 (0.000)	1837.187 (0.000)
Df	476	477
CFI	0.834	0.908
RMSEA	0.069 (0.065–0.072)	0.044 (0.041–0.047)

To improve model fit, a trimmed model was estimated by removing the statistically insignificant direct path between personal–social discrimination and help-seeking. As shown in Table 6, the fit indices indicate that the trimmed model provides a more streamlined and statistically robust representation of the data. The CFI increased from 0.834 to 0.908, and the RMSEA decreased from 0.069 to 0.044, highlighting the model’s better alignment with the observed data. A chi-square difference test comparing the full path model (χ^2^(476) = 1839.968, p < 0.001) and the trimmed model (χ^2^(477) = 1837.187, p < 0.001) showed no significant difference between the two models (Δχ^2^(1) = 2.781, p = 0.095). Given the improved fit and the maintenance of theoretical integrity, the trimmed model was selected for final interpretation.

**Fig. 2:**
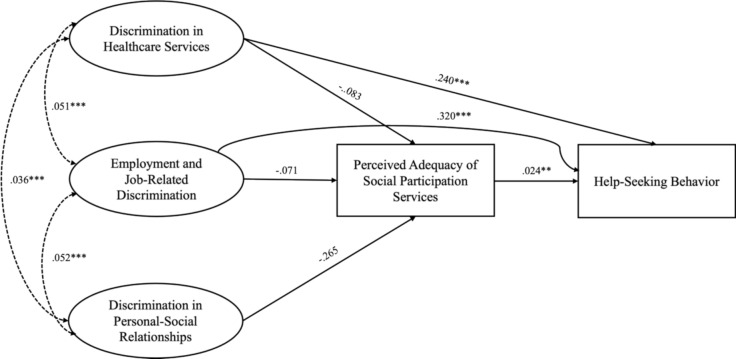
Trimmed model of the relationship between discrimination, help-seeking behaviors, and the perceived adequacy of social participation services. *Regression paths are represented by straight lines with one arrowhead, while covariances are shown as dotted lines with two arrowheads

As depicted in Fig. 2, healthcare and employment discrimination had significant positive direct effects on help-seeking behavior, suggesting that individuals facing discrimination in these areas are more likely to seek help. Overall, the findings indicate that there is no mediating effect of perceived adequacy of social participation services on the relationship between discrimination experiences and help-seeking behavior. Instead, the perceived adequacy of social participation services positively influenced help-seeking behavior, implying that individuals who perceive these services as highly sufficient are more inclined to seek help.

To examine the statistical significance and magnitude of the direct, indirect, and total effects of the discrimination domains on help-seeking behavior, mediated by perceived adequacy of social participation services, effect decomposition was conducted based on the trimmed model (see Table 7). The findings show that both discrimination in healthcare services and job- or employment-related discrimination exhibit statistically significant direct and total effects, as zero does not fall within their 95% confidence intervals. However, the indirect effects for these variables were not statistically significant, suggesting the absence of any mediation effects. Furthermore, the effect of discrimination in personal–social relationships on help-seeking was not statistically significant for either the indirect or total effects.

**Table 7:** Effect decomposition table

Predictor	Direct effect (95% CI)	Indirect effect (95% CI)	Total effect (95% CI)
Discrimination in Healthcare Services	0.240 (0.102, 0.424)	− 0.002 (− 0.009, 0.003)	0.238 (0.099, 0.422)
Employment and Job-Related Discrimination	0.320 (0.224, 0.406)	− 0.002 (− 0.015, 0.010)	0.318 (0.221, 0.403)
Discrimination in Personal–Social Relationships		− 0.007 (− 0.034, 0.010)	− 0.007 (− 0.034, 0.010)

## Discussion

The objective of this study was to understand the extent to which different forms of discrimination impact help-seeking behavior and to assess whether the perceived adequacy of social participation services mediates this relationship. By exploring these pathways, the study sought to provide insights into the mechanisms by which discrimination affects the coping behaviors of individuals with serious mental illness and whether social support services can potentially buffer or mediate this impact. The findings showed that discrimination in healthcare and employment settings had significant positive direct effects on help-seeking behavior, while discrimination in personal–social relationships did not. Contrary to the hypothesis that greater discrimination would decrease help-seeking, the significant effects of healthcare discrimination and employment discrimination suggest that individuals with serious mental illness who face discrimination in these critical domains are more likely to seek help. This challenges previous literature, which often associates higher levels of discrimination with increased reliance on emotion-focused coping strategies, such as avoidance (Brittian et al., 2013; Compas, 1995; Scott & House, 2005).

This discrepancy can be explained by the characteristics of the study participants, who are users of community-based mental health services and may have developed more active, problem-focused coping mechanisms through their exposure to structured mental health rehabilitation programs. Building on Bandura’s (1977) social learning theory, the direct reinforcement and peer modeling processes within mental health rehabilitation facilities may equip individuals with active coping skills, such as help-seeking, when they confront discrimination. This interpretation aligns with the observed positive relationship between healthcare and employment discrimination and help-seeking behavior. However, the absence of a significant effect for personal–social discrimination warrants further exploration. Discrimination in personal–social relationships may be perceived as more personal and perhaps less amenable to formal interventions, such as workplace policies or healthcare systems through external help-seeking channels. This may be because these contexts often involve personal connections rather than institutional structures. This finding could reflect a different coping process where interpersonal discrimination is managed through informal networks or internal coping strategies rather than external help-seeking behavior.

Moreover, the findings suggest that social participation services did not mediate the relationship between discrimination and help-seeking behavior, which contradicts the initial hypothesis of this study. However, the direct path was significant, indicating that when individuals perceive these services as adequate, they are more likely to engage in help-seeking behaviors. This aligns with Lazarus and Folkman’s (1984) stress appraisal-coping theory, which posits that problem-focused coping arises when individuals perceive control over stressors. This theory posits that individuals are more inclined to engage in problem-focused coping when they perceive greater control over their environment. In other words, social participation services may empower individuals with serious mental illness to seek help by providing additional resources and enhancing individuals’ perceived control, thereby increasing help-seeking behaviors.

Nevertheless, the absence of a mediating role for the perceived adequacy of social participation services in the relationship between discrimination and help-seeking behaviors may reflect the characteristics of these services in Korea. From the perspective of the stress appraisal-coping model (1984), the lack of mediation suggests that these services may not yet be sufficiently integrated into individuals’ cognitive appraisals of their coping resources. Given that social participation services for this population are still in their early stages in Korea, it is plausible that individuals may seek help directly in response to discrimination rather than relying on these services. For instance, Ha (2021) emphasized the need for stable material support for peer-led organizations, which have been emerging since the 2010s as one representative type of social participation service for people with serious mental illness in Korea and called for the establishment of a stronger institutional foundation for such support. Thus, it can be inferred that social participation services for people with serious mental illness in Korea remain in their infancy, and as a result, these services may not yet be effective or widely recognized by individuals with mental illness.

An alternative explanation for the lack of a mediating effect is that individuals may turn to social participation services not as a proactive strategy to improve access to healthcare or employment, but rather as a compensatory resource when excluded from formal systems. Social participation services, such as peer-led support groups and self-help programs, often arise in response to systemic barriers in formal institutions. They provide non-judgmental, empowering environments that contrast with the disempowerment individuals may experience in formal settings, offering opportunities like volunteer work, skill-building, and employment support outside traditional frameworks (Giummarra et al., 2022; Webber & Fendt-Newlin, 2017). As a result, rather than acting as a bridge to reintegrate individuals into formal systems, these services may primarily fill gaps for those who feel alienated from mainstream domains of employment and healthcare. In this context, social participation services could function as parallel support systems rather than pathways back to formal healthcare or employment, particularly when systemic exclusion persists.

In conclusion, this study reveals that discrimination in healthcare and employment settings significantly drives help-seeking behavior among individuals with serious mental illness. This aligns with the stress appraisal-coping model, which highlights the importance of cognitive appraisal and resource availability in shaping coping responses. This finding suggests that participants, many of whom are engaged in community-based mental health services, may have developed active coping strategies like help-seeking in response to discrimination. However, personal–social discrimination did not show a similar effect, likely because it is perceived as more personal and less actionable through formal channels. While the adequacy of social participation services did not mediate the relationship between discrimination and help-seeking, it still had a direct positive influence, indicating that individuals who perceive these services as sufficient are more likely to seek help. The lack of a mediating effect may reflect the early-stage development of these services in South Korea, where they are not yet fully established or perceived as effective. Overall, the findings underscore the importance of institutional support in healthcare and employment contexts and the need to further develop social participation services to better support help-seeking behaviors for those with serious mental illness.

### Implications for Practice

The findings of the study suggest several important implications. First, it is essential to expand the range of social participation services available to people with serious mental illness. This includes developing peer support services, as well as cultural, artistic, leisure, and physical activity programs. Previous literature highlights that mental health services supporting social participation and community engagement can yield positive outcomes such as self-management, a sense of belonging, and empowerment (Moon et al., 2020). However, in Korea, social participation services are currently scarce and lack diversity (National Human Rights Commission of Korea, 2021). Importantly, the findings suggest that individuals may turn to social participation services as compensatory mechanisms when they feel excluded from formal healthcare and employment settings. This highlights the need to strengthen these services to better support individuals who have experienced discrimination, ensuring they offer not only social and emotional support, but also resources that address the challenges of exclusion. To address this gap, there is a clear need to diversify these programs, incorporating activities that align with the specific needs of individuals with serious mental illness and offering them broader opportunities for meaningful social engagement.

Second, to make these expanded services effective, practitioners and policymakers should prioritize improving the visibility and accessibility of social participation services. Individuals with serious mental illness may not yet recognize these services as valuable resources, so it is vital to promote public awareness and credibility through community outreach, educational campaigns, and collaboration with mental health organizations. Such efforts can help foster greater understanding and utilization of these services by individuals with serious mental illness who might benefit from them.

Third, there is a pressing need to increase the number and equitable distribution of mental health rehabilitation facilities across South Korea. These facilities are key providers of social participation services within the Korean mental health system (National Human Rights Commission of Korea, 2020). However, as noted by the National Human Rights Commission of Korea (2020), mental health rehabilitation facilities are not available in 45.9% of regions across the country. According to Ministry of Health and Welfare of South Korea (2023), the number of these facilities increased from 348 in 2018 to 349 as of June 2022, reflecting a mere addition of one facility over a five year period (Ministry of Health and Welfare of South Korea, 2023). As such, people with serious mental illness who reside in areas without such facilities are unable to access social participation services. To address this disparity, it is essential to expand their number and ensure their more equitable distribution across the country.

### Study Limitations and Recommendations for Future Research

The findings of this study should be understood considering the following limitations. First, the cross-sectional design limits the ability to infer causality. Future research could benefit from utilizing a longitudinal design, which would allow for the examination of temporal relationships and provide stronger evidence of causality.

Second, this study utilized secondary data from a survey conducted by the National Human Rights Commission of Korea, which was not specifically designed for the purposes of this research and lacked validated measures. For instance, help-seeking behavior was measured only by whether individuals sought help in a discriminatory situation, without distinguishing between informal and formal sources, potentially obscuring important patterns. Additionally, self-reported data may introduce biases, such as social desirability or recall bias. Future research should employ objective measurements and precise operational definitions. For example, examining factors related to availability of social participation services, such as local government budgets and staffing, could offer deeper insights into structural factors influencing help-seeking and social participation.

Third, the study sample consisted only of individuals who had utilized community-based mental health rehabilitation facilities, potentially introducing sampling bias. Considering that only 12.1% of individuals with serious mental illness in Korea utilize mental health services (National Center for Mental Health, 2023), this may result in a more optimistic portrayal of help-seeking behavior and service engagement compared to the broader population of individuals with mental illness, particularly those who face barriers to accessing services or choose not to seek help. Consequently, the findings may not fully generalize to the entire population of individuals with mental illness in Korea. Future research should examine the experiences of individuals who do not use services to better understand barriers to service access and provide a more comprehensive understanding of help-seeking behavior.

Fourth, data collection took place in 2020 during the COVID-19 pandemic, which may have influenced participants’ perceptions of discrimination, social participation, and help-seeking, due to changes in service availability and increased isolation. These limitations emphasize the need for future research to adopt more robust measures and methodologies, incorporating objective data such as the availability of social participation services in Korean society, including local government budgets allocated to and staffing for these services.

Lastly, while this study relied on quantitative methods, future research could benefit from employing qualitative methodologies to gain a deeper understanding of the lived experiences of social exclusion faced by individuals with serious mental illness in South Korea and their pathway of turning to social participation services, and see whether it was related to feeling unwelcome or excluded from healthcare and employment spaces. Qualitative approaches could provide rich insights into how individuals perceive and navigate discrimination, as well as their motivations for turning to social participation services. Such methods would complement the current findings by uncovering subjective and contextual factors that may not be captured through quantitative measures, ultimately contributing to enhanced knowledge of the connections between discrimination, help-seeking behaviors, and social participation services.

## Conclusion

In conclusion, this study highlights the significant impact of perceived discrimination in healthcare and employment on help-seeking behaviors among individuals with serious mental illness in South Korea. It also emphasizes the direct influence of the perceived adequacy of social participation services on these behaviors. Although the study found no mediating role for social participation services, their direct impact remains critical in fostering help-seeking. The findings suggest that individuals with serious mental illness, especially those exposed to discrimination, may still rely on more established coping strategies rather than underdeveloped social participation services, which are still in their infancy in Korea.

The findings emphasize the need to view individuals with serious mental illness not as passive recipients of care, but as active agents in their recovery, capable of seeking solutions and coping with discrimination. This aligns with both the human rights and social models of disability, which frame recovery as a process driven by the individual’s capacity for agency and resilience, even in the face of discrimination. By enhancing the availability and visibility of social participation services, mental health systems can better support individuals in becoming active participants in their recovery.
